# Total Saponins of Radix Clematis Regulate Fibroblast-Like Synoviocyte Proliferation in Rheumatoid Arthritis via the LncRNA OIP5-AS1/MiR-410-3p/Wnt7b Signaling Pathway

**DOI:** 10.1155/2022/8393949

**Published:** 2022-05-27

**Authors:** Lingyu Pan, Yuan Sun, Hui Jiang, Yan Chen, Yeke Jiang, Yanquan Han, Yongzhong Wang

**Affiliations:** ^1^The First Affiliated Hospital of Anhui University of Chinese Medicine, Hefei 230031, Anhui, China; ^2^Anhui University of Chinese Medicine, Hefei 230031, Anhui, China

## Abstract

**Background:**

Rheumatoid arthritis (RA) is the most common autoimmune disease and affects multiple joints. Previous studies have shown that total saponins of *Radix clematidis* (TSC) have a clear therapeutic effect on RA, but the specific mechanism has not yet been clarified. Literature screening and previous research suggest that the lncRNA OIP5-AS1/miR-410-3p/Wnt7b signaling pathway exerts a regulatory effect on the pathogenesis of RA. In this study, we examined whether the TSC treatment of RA affects the lncRNA OIP5-AS1/miR-410-3p/Wnt7b pathway.

**Materials and Methods:**

Freund's complete adjuvant was used to create an adjuvant arthritis (AA) rat model with rat synovial cells being harvested and cultured. The experiment comprises a normal group, model group, TSC optimal-dose group, TSC optimal-dose group + lncRNA OIP5-AS1siRNA group, lncRNA OIP5-AS1 siRNA group, and lncRNA OIP5-AS1 siRNA + NC group. MMT was used to screen the optimal concentration of TSC. The level of lncRNA OIP5-AS1, miR-410-3p, Wnt7b, *β*-catenin, c-Myc, cyclin D1, GSK-3*β*, and SFRP4 mRNA were detected by real-time-qPCR, the expression of Wnt7b, *β*-catenin, c-Myc, cyclin D1, GSK-3*β*, and p-GSK-3*β* (Ser9) protein were detected by immunofluorescence and Western blot.

**Results:**

We found that TSC inhibits the proliferation of RA FLS, TSC significantly reduced lncRNA OIP5-AS1, Wnt7b, *β*-catenin, c-Myc, cyclin D1, and p-GSK-3*β*/GSK-3*β* mRNA/protein expression, whereas the miR-410-3p and SFRP4 mRNA/protein expression levels were significantly upregulated. Our data suggest that TSC can inhibit the excessive proliferation of FLS to treat RA, the mechanism of which may be closely related to regulation of the lncRNA OIP5-AS1/miR-410-3p /Wnt7b signaling axis and the Wnt signaling pathway.

## 1. Introduction

Rheumatoid arthritis (RA) is a chronic autoimmune-mediated inflammatory disease characterized by persistent synovitis, systemic inflammation, and joint destruction [1]. When inflammation occurs, the excessive proliferation and abnormal activation of fibroblast-like synoviocytes (FLS) in the synovial tissue promotes the production of pannus in the synovium. FLS can also secrete variety of proinflammatory cytokines that induce the infiltration of inflammatory cells and ultimately lead to progressive joint destruction and dysfunction, such as interleukin (IL) 6, IL-1*β*, and tumor necrosis factor-*α* (TNF-*α*) [2, 3]. The hyperproliferation of FLS plays a key role in the pathogenesis of RA.

Previous studies have shown that an imbalance between excessive proliferation and apoptosis of FLS is the pathological basis of RA [4]. The continuous activation of FLS when inflammation occurs can lead to tumorlike hyperplasia and the release of inflammatory factors, resulting in damage to articular cartilage [5]. Therefore, inhibiting the excessive activation of FLS is a key means of treating RA.

Long noncoding RNAs (lncRNAs) are nonprotein-coding RNA transcripts with a length >200 nucleotides [6]. LncRNAs participate in a wide range of biological and pathological processes and play important roles in cell growth and development. LncRNAs can regulate cell proliferation, differentiation, and apoptosis, thereby mediating the inflammation and immune processes of various diseases [7]. LncRNA OIP5-AS1 is ubiquitous in mammals. It is highly conserved during the evolution of vertebrates and highly expressed in many diseases [8, 9]. LncRNA OIP5-AS1 was shown to act as a ceRNA to regulate miRNAs and mediate cell proliferation, invasion, and migration in many diseases, such as osteosarcoma, gastric cancer, and hemangioma1 [10, 11]. However, whether lncRNA OIP5-AS1 regulates biological functions in RA, this has not been reported. Our earlier bioinformatics analyses indicated that lncRNA OIP5-AS1 is the key differential lncRNA in the development of RA, and miR-410-3p is the targeted miRNA of lncRNA OIP5-AS1. miR-410-3p was confirmed to play an important anti-inflammatory role in the pathogenesis of RA and regulate cell proliferation by acting on downstream target genes [2, 3]. Wnt7b is the common target gene of lncRNA OIP5-AS1 and miR-410-3p, and it is an important agonist in the Wnt classic signaling pathway [12]. The Wnt signaling pathway, an important mediator of RA, regulates the processes of cell differentiation, proliferation, migration, and invasion. Therefore, we chose the lncRNA OIP5-AS1/miR-410-3p/Wnt7b signaling pathway as the target of this study.

*Clematis chinensis* is composed of the dried roots and rhizomes of *Clematis chinensis* Osbeck, a plant in the Ranunculaceae family. It has the effect of curing rheumatism, promoting blood circulation, and eliminating blood stasis [13]. Total saponins of *Radix clematidis* (TSC) is the primary component responsible for the antiarthritis effects of *Clematis chinensis*.

In previous studies using an adjuvant arthritis (AA) rat model, we confirmed that TSC has a clear anti-inflammatory effect, and its therapeutic mechanism may be related to regulation of the JAK2/STAT3 signaling pathway and regulation of lipid, amino acid, and energy metabolism [14]. Therefore, in this study, we targeted the lncRNA OIP5-AS1/miR-410-3p/Wnt7b signaling pathway to study the mechanism of action of TSC in the treatment of RA.

## 2. Materials and Methods

### 2.1. Model Preparation

In this study, Sprague Dawley rats (male, SPF) were purchased from Anhui Experimental Animal Center. According to the method described in the previous study, rats were reared adaptively for 1 week. Except for the normal group, 0.1 mL of Freund's complete adjuvant (Sigma, 1001646446) was intradermally injected into the left hind toe of each rat. After 18 hours of modeling, the inflamed part began to swell. After about 10 days of modeling, rats developed secondary lesions. The paws on the opposite side of the inflamed side of rats began to swell, and knots appeared on the ears and tails of rats. The peak period of inflammation occurred 20 days after modeling [15]. Therefore, we anesthetized rats with 60 mg/kg pentobarbital on the 20th day after modeling. At the end of the experiment, rats were sacrificed via exsanguination under anesthesia. All animal experiments were approved by the Animal Ethics Committee of the Anhui University of Chinese Medicine (license number: LLSC20160336).

The hind limbs of rats were separated, and synovial layer tissue was removed. FLS were cultured in complete Dulbecco's Modified Eagle Medium with 20% fetal bovine serum (Gibco, 1715752) at 37°C and 5% CO_2_ using the tissue explant method. Under an inverted fluorescence microscope, the cells swimming from the edge of the tissue block were observed, and the morphology and characteristics of primary and passaged synovial cells were recorded. Vimentin was identified using an immunofluorescence staining kit according to the manufacturer's instructions [16, 17].

### 2.2. MTT Assay

The 3-(4,5-dimethylthiazol-2-yl)-2,5-diphenyltetrazolium bromide (MTT) assay was used to determine the effect of different concentrations of TSC on the proliferation of synovial cells at 24 and 48 h after exposure. Rats were divided into normal, model, and different TSC concentration groups (0.5, 2.5, 12.5, 62.5, 312.5, and 1562.5 *μ*g/mL). Synovial cells in the logarithmic growth phase were inoculated into a 96-well culture plate and incubated for approximately 48 h. The cells were then observed under an inverted fluorescence microscope to determine when they reach ≥80% confluence. Preprepared concentrations of TSC were then added to the wells, and the cells were incubated for another 24 or 48 h. After culture, MTT solution was added to each well (5 mg/mL, 20 *μ*L) and incubated for 4 h. Next, 200 *μ*L of dimethyl sulfoxide was added to each well, and the plate was shaken at room temperature for 10 min to allow the reagent to dissolve. The absorbance of each well was then recorded at 490 nm using a microplate reader (BioTek) to determine the effect of different concentrations of TSC on FLS proliferation.

### 2.3. Experiment Grouping

The experimental animals were divided into a normal group, model group, TSC optimal dose group, TSC optimal dose + lncRNA OIP5-AS1siRNA group, lncRNA OIP5-AS1 siRNA group, and lncRNA OIP5-AS1 siRNA + NC group, and after 48 h of treatment, the cells were collected.

### 2.4. Cell Transfection

To suppress lncRNA OIP5-AS1, specific small interfering RNAs (siRNAs) targeting lncRNA OIP5-AS1 were synthesized and purchased from Hefei Delpu Biotechnology Co., Ltd. (Hefei, China). Sequences of the constructed lncRNA OIP5-AS1 interference fragments are shown in Table 1. When cells grew to 40% confluence, Lipofectamine™ 2000 transfection reagent (Invitrogen) was used for siRNA transfection into FLS. At 48 h posttransfection, FLS were harvested, and the expression of lncRNA OIP5-AS1 was measured using RT-qPCR.

### 2.5. Quantitative Real-Time PCR (RT-qPCR)

TRIzol (Invitrogen, 90802) was used to extract total RNA from FLS according to the manufacturer's instructions. A total of 2 *μ*g of total RNA was used as a template for reverse transcription to synthesize cDNA (ABcolnal, RK20403). Quantitative fluorescent PCR (Thermo, USA) was used to determine the relative expression of lncRNA OIP5-AS1, miR-410-3p, Wnt7b, *β*-catenin, c-Myc, cyclin D1, GSK-3*β*, and SFRP4 mRNAs. The poly(A) tailing method was used for miR-410-3p tailing and cDNA synthesis. The primers used are listed in Table 2.

The downstream primers for miR-410-3p were directly provided in the miRNA tailing kit.

### 2.6. Immunofluorescence

FLS were fixed in 4% paraformaldehyde for 20 min, after which 0.5% Triton X-100 diluted in phosphate-buffered saline (PBS, Gibco, 8118311) was added to the wells and incubated for 20 min. Sections were rinsed three times with PBS, and 5% bovine serum albumin was added to these sections and blocked for 30 min. Diluted primary antibody: Wnt7b (Abcam, GR3241134-2 1 : 500); *β*-catenin (Proteintech, 00077341, 1 : 200); c-Myc (Proteintech, 00033258, 1 : 50); cyclin D1 (Abcam, GR197045-1, 1 : 50), p-GSK-3*β*(Ser9) (CST, #5588, 1 : 400); SFRP4 (Wuhan Aibotek Biotechnology Co., Ltd., 9100014210, 1 : 500) was added, and samples were placed in a humid box and incubated overnight at 4°C in the dark. Then, the corresponding second antibody was added and incubated at room temperature for 50 min. DAPI (Beijing Soleibo Technology Co., Ltd., 20181120) was used to stain the cell nucleus.

### 2.7. Western Blotting

FLS were washed with cold PBS three times and disrupted to prepare 100 *μ*L of protein lysate. Sodium dodecyl sulfate-polyacrylamide gel electrophoresis was used to separate total proteins, which were then transferred onto polyvinylidene fluoride (PVDF) membranes, which were blocked in TBST (washed twice with 1 × TBST for 2 min, then sealed with 5% skim milk). PVDF membranes were then incubated overnight at 4°C with the following primary antibodies: *β*-actin (1 : 50000); p-Wnt7b (1 : 5000); p-*β*-catenin (1 : 6000); p-c-Myc (1 : 2000); p-cyclin D1 (1 : 1000); p-GSK-3*β* (Ser9) (1 : 1000); and p-SFRP4 (1 : 500). The membranes were then washed three times with TBST and incubated for 2 h at room temperature with secondary antimouse or antirabbit antibody conjugated with horseradish peroxidase (1 : 10000). Proteins were detected using an enhanced chemiluminescence kit. Each protein band shown was representative of three replicates. Western blotting data were quantified using an ImageJ software.

### 2.8. Statistical Analysis

All data were analyzed using SPSS version 23.0, and data are shown as mean ± standard deviation. One-way analysis of variance was used for comparisons between the multiple groups, and the *t*-test was used for comparisons between the two groups. Differences were considered statistically significant at *P* < 0.05.

## 3. Results

### 3.1. TSC Inhibits the Proliferation of RA FLS

**Figure 1:**
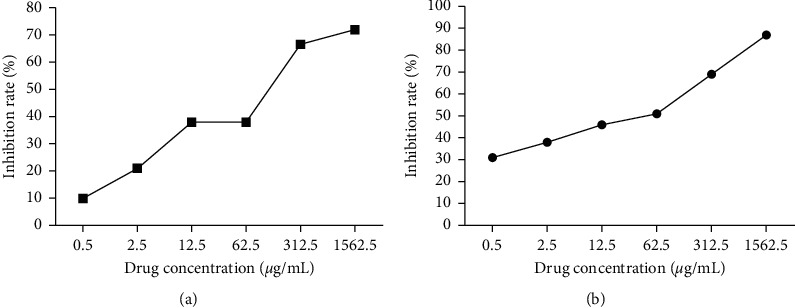
Inhibitory effect of TSC at different times (24 h/48 h on the growth of FLS of AA rats). (a) The rate of TSC inhibition of FLS proliferation at 24 h. (b) The rate of TSC inhibition of FLS proliferation at 48 h.

The MTT assay was used to evaluate the effect of TSC on the proliferation of FLS at different times of action and different drug concentrations. As shown in Figure 1, when the action time was 48 h, the effect of TSC on inhibiting the proliferation of synovial cells increased steadily, reaching a 50% inhibition at 62.5 *μ*g/mL. Therefore, in the follow-up experiment, the action time of the drug was selected as 48 h, and 62.5 *μ*g/mL was selected as optimal drug concentration.

### 3.2. Effect of TSC on the Expression of LncRNA OIP5-AS1 and MiR-410-3p mRNAs in FLS

**Figure 2:**
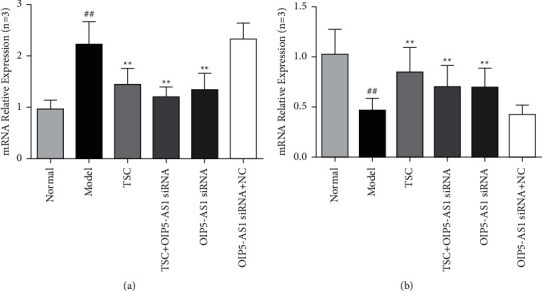
Effects of TSC intervention on mRNA expression of lncRNA OIP5-AS1 and miR-410-3p in FLS. (a) The effect of TSC intervention on the mRNA expression of lncRNA OIP5-AS1in FLS. (b) The effect of TSC intervention on the mRNA expression of miR-410-3p in FLS. ^##^*P* < 0.01 compared with the normal group; ^*∗∗*^*P* < 0.01 compared with the model group.

Compared with the normal group, the mRNA expression level of lncRNA OIP5-AS1 in the model group and the OIP5-AS1/Wnt7b siRNA + NC group was significantly increased, and the mRNA expression level of miR-410-3p was significantly reduced (*P* < 0.01). Compared with the model group, the TSC + lncRNA OIP5-AS1 siRNA optimal-dose group and the lncRNA OIP5-AS1 siRNA group, the expression level of lncRNA OIP5-AS1 mRNA decreased significantly, and the expression of miR-410-3p mRNA increased significantly. These differences were statistically significant (*P* < 0.01). Compared with the model group, there were no significant changes in the mRNA expression of lncRNA OIP5-AS1 or miR-410-3p in the lncRNA OIP5-AS1 siRNA + NC group (Figure 2).

### 3.3. Effect of TSC on the Expression of Wnt7b/*β*-Catenin Signaling Pathway Components in FLS

**Figure 3:**
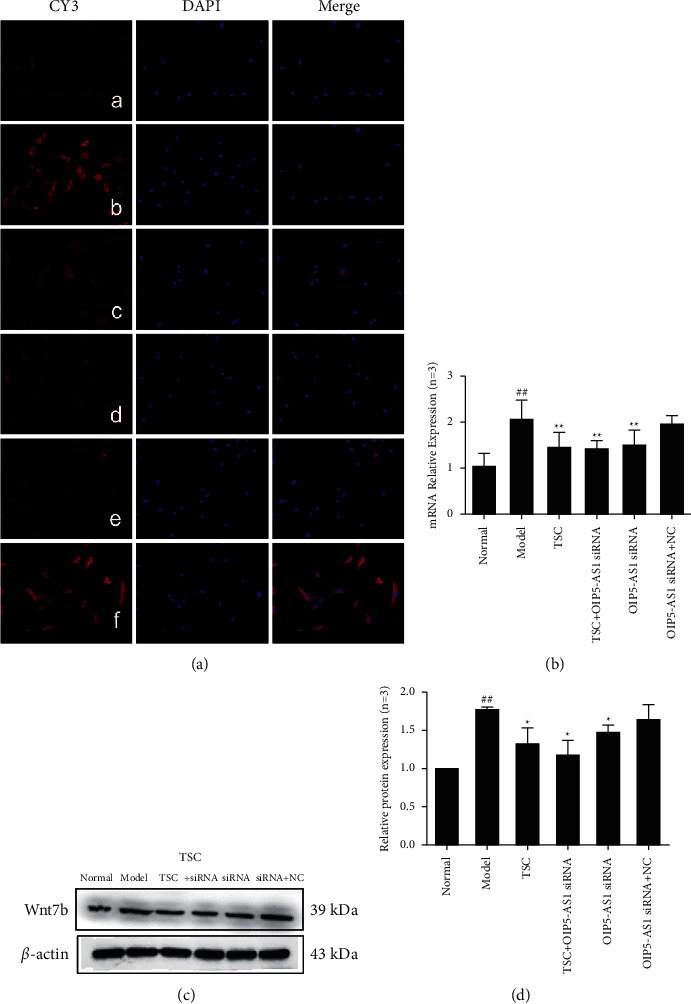
Effect of TSC on the expression of Wnt7b protein and mRNA in FLS of AA rats. (a) The change in Wnt7b protein expression was observed by immunofluorescence analysis (×200): (A) normal group, (B) model group, (C) TSC group, (D) TSC + lncRNA OIP5-AS1 siRNA group, (E) lncRNA OIP5-AS1 siRNA group, and (F) lncRNA OIP5-AS1 siRNA + NC group. (b) The change in Wnt7b mRNA expression was observed by RT-qPCR. (c) The change in Wnt7b protein expression was observed by Western blotting. (d) Semiquantitative analysis of Wnt7b protein. ^##^*P* < 0.01 compared with the normal group; ^*∗*^*P* < 0.05 and ^*∗∗*^*P* < 0.01 compared with the model group.

**Figure 4:**
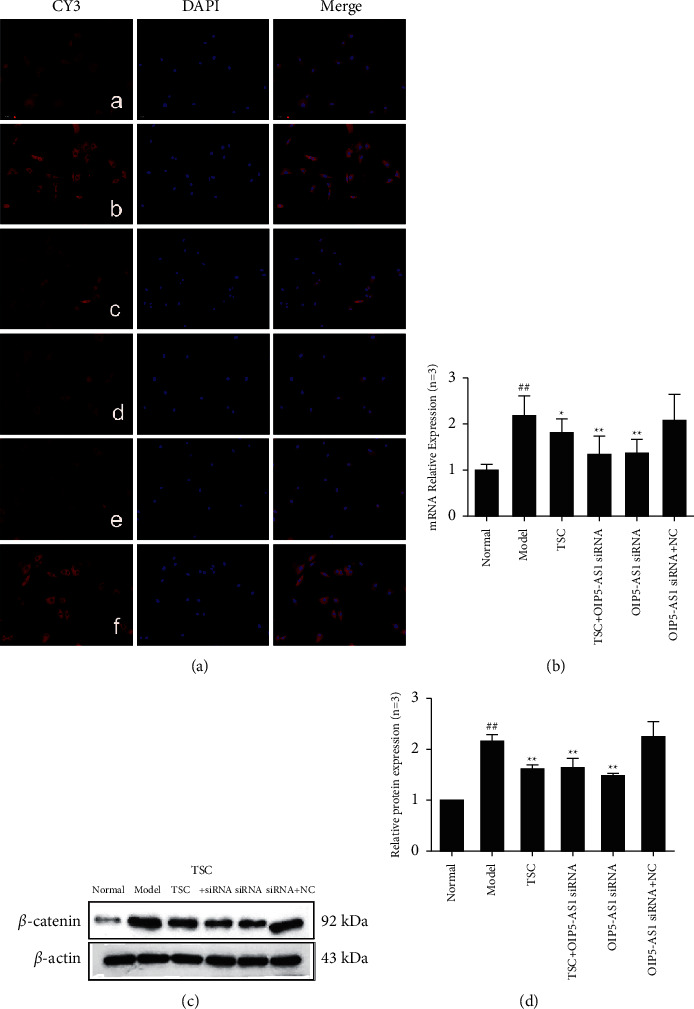
Effect of TSC on the expression of *β*-catenin protein and mRNA in FLS of AA rats. (a) The change in *β*-catenin protein expression was observed by immunofluorescence analysis (×200): (A) normal group, (B) model group, (C) TSC group, (D)TSC + lncRNA OIP5-AS1 siRNA group, (E) lncRNA OIP5-AS1 siRNA group, and (F) lncRNA OIP5-AS1 siRNA + NC group. (b) The change in *β*-catenin mRNA expression was observed by RT-qPCR. (c) The change in *β*-catenin protein expression was observed by Western blotting. (d) Semiquantitative analysis of *β*-catenin protein expression. ^##^*P* < 0.01 compared with the normal group; ^*∗*^*P* < 0.05 and ^*∗∗*^*P* < 0.01 compared with the model group.

**Figure 5:**
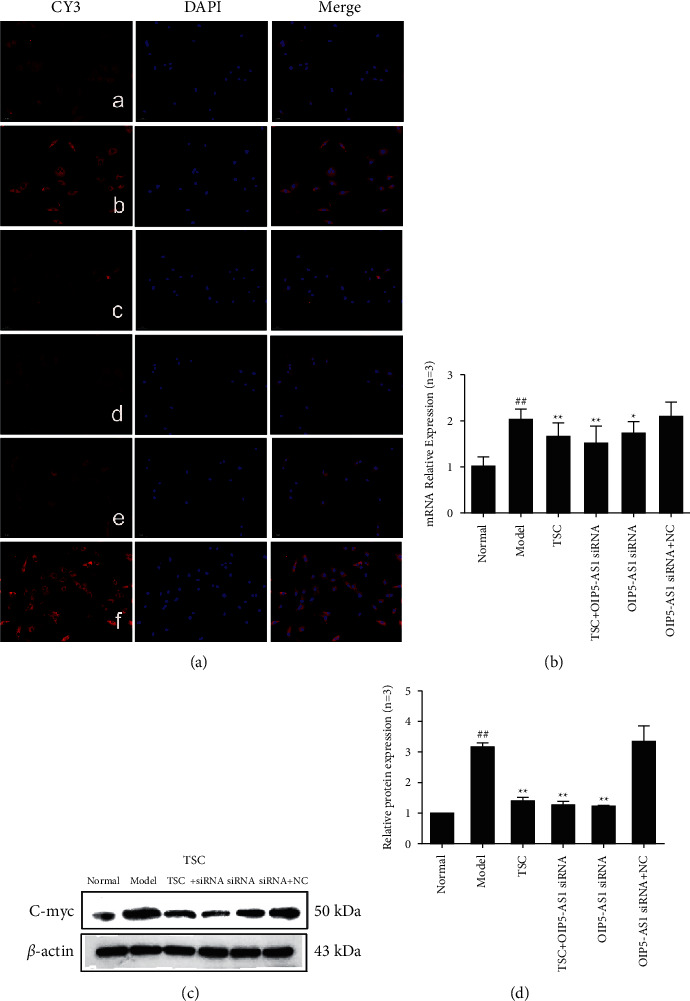
Effect of TSC on the expression of c-Myc protein and mRNA in FLS of AA rats. (a) The change in c-Myc protein expression was observed by immunofluorescence analysis (×200): (A) normal group, (B) model group, (C) TSC group, (D) TSC + lncRNA OIP5-AS1 siRNA group, (E) lncRNA OIP5-AS1 siRNA group, and (F) lncRNA OIP5-AS1 siRNA + NC group. (b) The change in c-Myc mRNA expression was observed by RT-qPCR. (c) The change in c-Myc protein expression was observed by Western blotting. (d) Semiquantitative analysis of c-Myc protein expression. ^##^*P* < 0.01 compared with the normal group; ^*∗*^*P* < 0.05 and ^*∗∗*^*P* < 0.01 compared with the model group.

**Figure 6:**
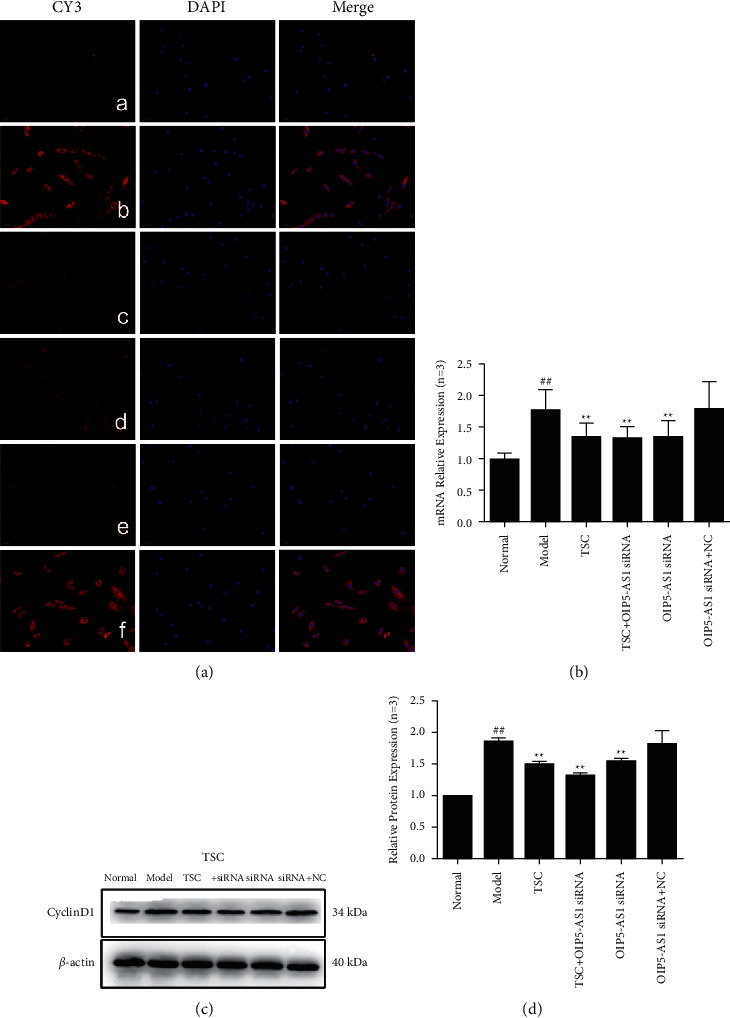
Effect of TSC on the expression of cyclin D1 protein and mRNA in FLS of AA rats. (a) The change in cyclin D1 protein expression was observed by immunofluorescence analysis (×200): (A) normal group, (B) model group, (C) TSC group, (D) TSC + lncRNA OIP5-AS1 siRNA group, (E) lncRNA OIP5-AS1 siRNA group, and (F) lncRNA OIP5-AS1 siRNA + NC group. (b) The change in cyclin D1 mRNA expression was observed by RT-qPCR. (c) The change in cyclin D1 protein expression was observed by Western blotting. (d): Semiquantitative analysis of cyclin D1 protein expression. ^##^*P* < 0.01 compared with the normal group; ^*∗*^*P* < 0.05 and ^*∗∗*^*P* < 0.01 compared with the model group.

**Figure 7:**
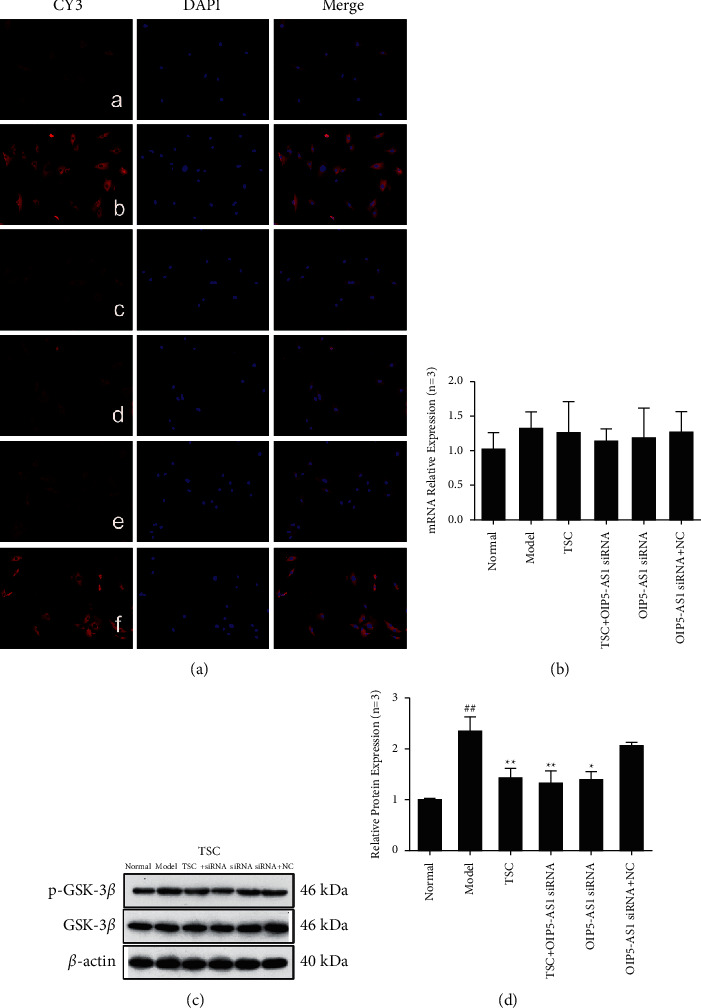
Effect of TSC on the expression of GSK-3*β*/p-GSK-3*β* protein and mRNA in FLS of AA rats. (a) The change in GSK-3*β*/p-GSK-3*β* protein expression was observed by immunofluorescence analysis (×200): (A) normal group, (B) model group, (C) TSC group, (D) TSC + lncRNA OIP5-AS1 siRNA group, (E) lncRNA OIP5-AS1 siRNA group, and (F) lncRNA OIP5-AS1 siRNA + NC group. (b) The change in GSK-3*β*/p-GSK-3*β* mRNA expression was observed by RT-qPCR. (c) The change in GSK-3*β*/p-GSK-3*β* protein expression was observed by Western blotting. (d) Semiquantitative analysis of GSK-3*β*/p-GSK-3*β* protein expression. ^##^*P* < 0.01 compared with the normal group; ^*∗*^*P* < 0.05 and ^*∗∗*^*P* < 0.01 compared with the model group.

The results of immunofluorescence experiments showed that Wnt7b, *β*-catenin, c-Myc, cyclin D1, GSK-3*β*, and p-GSK-3*β* (Ser9) were primarily expressed in the cytoplasm (Figures 3–7). RT-qPCR and Western blotting results showed that compared with the normal group, the expression of Wnt7b, *β*-catenin, c-Myc, cyclin D1, GSK-3*β*, and p-GSK-3*β* (Ser9) protein and mRNA in the model group and the OIP5-AS1 siRNA + NC group was higher. Compared with the model group, the protein and mRNA expression levels of Wnt7b, *β*-catenin, c-Myc, cyclin D1, GSK-3*β*, and p-GSK-3*β* (Ser9) in the TSC optimal-dose group, a TSC optimal dose + lncRNA OIP5-AS1 siRNA group, and a lncRNA OIP5-AS1 siRNA group were significantly reduced (*P* < 0.01). Compared with the model group, Wnt7b protein and mRNA expression levels in the lncRNA OIP5-AS1 siRNA + NC group were not significantly different.

### 3.4. Effect of TSC on the Expression of SFRP4 in FLS

**Figure 8:**
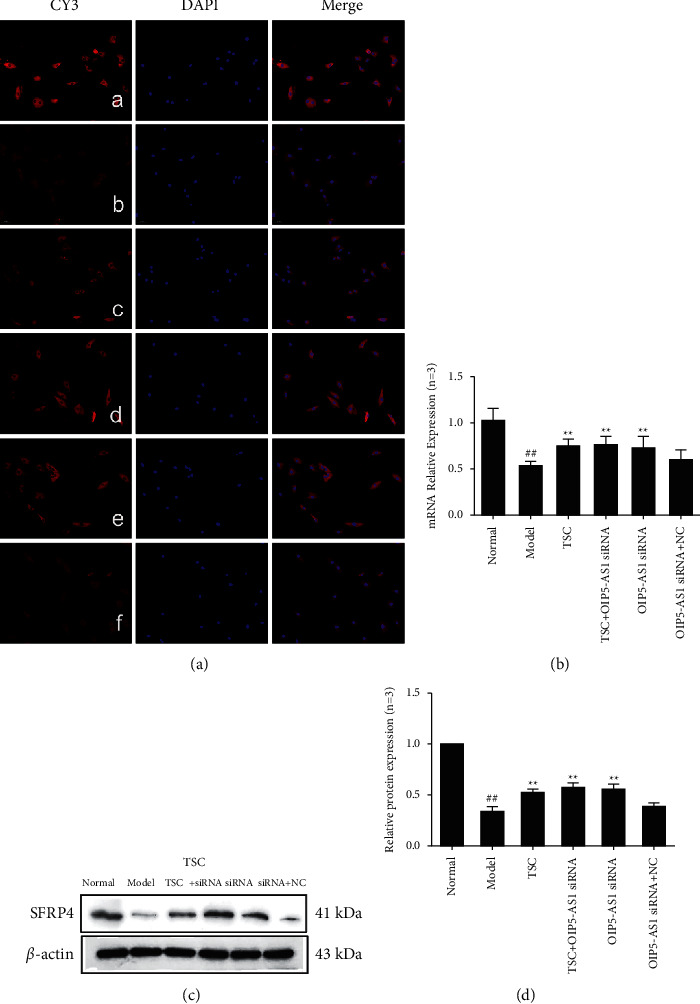
Effect of TSC on the expression of SFRP4 protein and mRNA in FLS of AA rats. (a) The change in SFRP4 protein expression was observed by immunofluorescence analysis (×200): (A) normal group, (B) model group, (C) TSC group, (D) TSC + lncRNA OIP5-AS1 siRNA group, (E) lncRNA OIP5-AS1 siRNA group, and (F) the lncRNA OIP5-AS1 siRNA + NC group. (b) The change in SFRP4 mRNA expression was observed by RT-qPCR. (c) The change in SFRP4 protein expression was observed by Western blotting. (d) Semiquantitative analysis of SFRP4 protein expression. ^##^*P* < 0.01 compared with the normal group; ^*∗*^*P* < 0.05 and ^*∗∗*^*P* < 0.01 compared with the model group.

The results of immunofluorescence analyses showed that SFRP4 was primarily expressed in the cytoplasm (Figure 8). RT-qPCR and Western blotting results showed that compared with the normal group, the protein and mRNA expression levels of SFRP4 in the model group and the OIP5-AS1 siRNA + NC group were low. Compared with the model group, the optimal TSC dose group, the optimal TSC dose + lncRNA OIP5-AS1 siRNA group, and the lncRNA OIP5-AS1 siRNA group, SFRP4 protein and mRNA expression levels were significantly increased (*P* < 0.01). Compared with the model group, there was no significant change in the protein and mRNA expression levels of SFRP4 in the lncRNA OIP5-AS1 siRNA + NC group.

## 4. Discussion

RA is a systemic chronic autoimmune disease, and its etiology is complex and affects multiple systems. RA is characterized by synovial inflammation, which can cause gradual destruction of bone and cartilage and lead to disability in severe cases [18]. The complications of RA can be serious with pathological features, including hyperproliferation of synovial tissue cells, formation of pannus, and cartilage erosion. The typical clinical features are joint swelling, pain, morning stiffness, and symmetry, often accompanied by involvement of joints and other organs [19]. In RA, the joint synovium is divided into a synovial lining layer and a subsynovial layer. The two types of main cells in the synovial lining layer are macrophage-like synoviocytes and FLS, which are referred to as type A and type B synovial cells, respectively. FLS are also known as synovial lining fibroblasts, which are key effector cells in the synovium and an important part of synovial vascular proliferation tissue [4, 20].

LncRNA not only regulates cell proliferation, differentiation, and apoptosis but also mediates inflammation and immune processes of various diseases and plays an important role in the process of cell growth and development [7, 21]. LncRNA OIP5-AS1 is ubiquitous in mammals. In many diseases, such as osteosarcoma, gastric cancer [9], and hemangioma, lncRNA OIP5-AS1 act as ceRNA that regulates miRNAs and mediates cell proliferation, invasion, and migration [10, 11]. Based on target prediction, Yang confirmed that lncRNA OIP5-AS1 is a molecular sponge that regulates miR-410, and the expression of lncRNA OIP5-AS1 and miR-410 is negatively correlated [22]. In the present study, the mRNA level of the lncRNA OIP5-AS1 in the TSC group, the TSC + lncRNA OIP5-AS1siRNA group, and the lncRNA OIP5-AS1siRNA group was significantly downregulated compared with the model group, whereas the mRNA level of miR-410-3p was significantly upregulated. The protein and mRNA expression of the target gene Wnt7b was also significantly downregulated.

miRNAs are short, noncoding, single-stranded RNAs that play important roles in the pathology and biology of many diseases. miRNAs are posttranscriptional regulators of gene expression and have been widely studied as oncogenes in variety of cancers [23]. Many studies have shown that miR-410-3p functions as a tumor suppressor gene in breast cancer, pancreatic cancer, glioma, and other diseases and primarily inhibits cell proliferation [24]. The expression of miR-410-3p is downregulated in certain cancers. Overexpression of miR-410-3p significantly inhibits cancer cell invasion and cell proliferation. A recent study showed that miR-410-3p is involved in the process of RA inflammation and regulates cell proliferation and apoptosis [2]. The expression of miR-410-3p is low in human RA tissues and cells. Upregulating miR-410-3p can reduce the expression of inflammatory cytokines such as TNF-*α* and IL-6 and promote the proliferation of FLS while inhibiting apoptosis [3]. Many reports have confirmed the interaction between lncRNA OIP5-AS1 and miR-410-3p. In myeloma and glioma, both LNC and MIR are negatively regulated [25]. In this study, we found that TSC inhibits the expression of LNC in FLS, increasing the level of MIR and regulating downstream signaling pathways to ameliorate the symptoms of RA.

The Wnt signaling pathway is an important regulator of cell growth and development, and the classic Wnt/*β*-catenin signaling pathway regulates cell proliferation, migration, invasion, and inflammation [26]. The Wnt signaling pathway plays an important role in RA inflammation, cartilage metabolism, regulation of FLS proliferation, apoptosis, and bone balance. *β*-Catenin is the key gene in the classical Wnt signaling pathway, playing a central role in signal transduction that is directly related to the abnormal proliferation of RA synovial cells. Activation of the Wnt signaling pathway can lead to an increase in the level of *β*-catenin in the synovium of RA patients, thereby promoting stable activation of the synovium of the joint [27, 28]. Yoshioka et al. [29] confirmed that *β*-catenin has a strong inhibitory effect on the proliferation of RA-FLS cells. C-Myc and cyclin D1 are direct downstream targets of the *β*-catenin/Wnt signaling pathway. C-Myc is a major proto-oncogene that regulates activation, transcription, and suppression of other genes [30]. Cyclin D1 is an important regulator of the G1 phase of the cell cycle. Overexpression of cyclin D1 shortens the cell cycle, thus promoting rapid cell proliferation.

GSK-3, a serine-threonine kinase with a structural activity, is involved in the regulation of multiple signaling pathways [31]. Two subtypes are currently known: GSK-3*α* and GSK-3*β*. GSK-3*β* is located upstream of the *β*-catenin/Wnt signaling pathway, and the level of *β*-catenin is negatively regulated by GSK-3*β*–mediated phosphorylation or other degradation pathways [32]. When Ser9 is phosphorylated, the activity of GSK-3*β* is inhibited, and the phosphorylation and degradation of *β*-catenin are blocked, thereby positively regulating the *β*-catenin/Wnt signaling pathway.

As a negative regulator of the Wnt/*β*-catenin pathway, SFRP attenuates Wnt signaling, antagonizing the Wnt signaling pathway [33]. This study found that drug intervention using TSC affects the expression of key factors downstream of the classic Wnt signaling pathway. Compared with the model group, the mRNA and protein expression levels of *β*-catenin, c-Myc, and cyclin D1 were significantly reduced after TSC drug intervention, whereas the mRNA and protein expression levels of the negative regulatory protein SFRP4 of the Wnt pathway were significantly increased. There was no significant change in the expression levels of GSK-3*β* among the groups, and the mRNA and protein expression levels of p-GSK-3*β* (Ser9)/GSK-3*β* were significantly reduced. In the TSC + lncRNA OIP5-AS1 siRNA and lncRNA OIP5-AS1 siRNA groups, the expression levels of key factors downstream of the Wnt pathway, including p-GSK-3*β* (Ser9)/GSK-3*β*, were significantly downregulated, whereas the expression of SFRP4 was significantly upregulated. These data suggest that downregulation of Wnt7b expression inhibits the expression of downstream *β*-catenin and also inhibits activation of the Wnt signaling pathway.

The adverse effects of RA lower the quality of patients' life and bring a heavy burden to the society. At present, main drugs for the treatment of RA include nonsteroidal anti-inflammatory drugs, immunosuppressants, and glucocorticoids, but all have certain side effects for a long-term use, so it is very urgent to find a new alternative. This study further explained the pathogenesis of RA, clarified the therapeutic effect of TSC on RA, provided experimental support for TSC in the treatment of RA patients, and provided new ideas for the treatment of RA. This study also enhances our confidence in the treatment of RA with traditional Chinese medicine.

## 5. Conclusion

TSC intervention significantly inhibited the proliferation of FLS. At the optimal dose, TSC significantly reduced the expression of lncRNA OIP5-AS1, the expression of miR-410-3p was significantly upregulated, and that of its target gene, Wnt7b, was significantly downregulated. The expression of key factors in the classic Wnt signaling pathway was also inhibited. Our data suggest that the inhibitory effect of TSC against the proliferation of FLS is related to regulation of the lncRNA OIP5-AS1/miR-410-3p/Wnt7b signaling axis and inhibition of the Wnt signaling pathway activation.

## Figures and Tables

**Table 1:** Sequences of OIP5-AS1 interference fragments.

Fragment	Sequence
siRNA1	Forward: 5′-UUUGUUGUUCUUAAACAACUU-3′
	Reverse: 5′-GUUGUUUAAGAACAACAAAAU-3′

siRNA2	Forward: 5′-AUCUAUUGCUUCAUUAUUGAU-3′
	Reverse: 5′-CAAUAAUGAAGCAAUAGAUAC-3′

siRNA3	Forward: 5′-AGCUAUUGGUUUAAGUAUCUA-3′
	Reverse: 5′-GAUACUUAAACCAAUAGCUUG-3′

**Table 2:** Primers used for real-time PCR.

Gene	Primers
LncRNA OIP5-AS1	Forward: 5′-AGTGCTGCGTTTTCTGCCCT-3′
	Reverse: 5′-CACCTCATACACTGGTGGCCC-3′

miR-410-3p	Forward: 5′-AATATAACACAGATGGCCTGT-3
	Downstream primers are directly provided by miRNA tailing kit

Wnt7b	Forward: 5′-GCGGTGATGGCGTATGT-3′
	Reverse: 5′-GGGCGCAGATGGGTATC-3′

*β*-Catenin	Forward: 5′-TATGAGTGGGAGCAAGGC-3′
	Reverse: 5′-CTGCGTGGATGGGATCT-3′

C-Myc	Forward: 5′-GGTCATCCCCATCAAGAG-3′
	Reverse: 5′-CATTTGCGGTTGTTGCT-3′

CyclinD1	Forward: 5′-GCGTACCCTGACACCAAT-3′
	Reverse: 5′-CTTCGCACTTCTGCTCCT-3′

GSK-3*β*	Forward: 5′-CTTTTCACAGGGCTACGC-3′
	Reverse: 5′-ACAGGCAAGCACATTTCC-3′

SFRP4	Forward: 5′-CCACACACCACTTGCCTT-3′
	Reverse: 5′-CACAGGCCACTCTGAACAC-3′

*β*-Actin	Forward: 5′-CCTCACTGTCCACCTTCCA-3′
	Reverse: 5′-GGGTGTAAAACGCAGCTCA-3′

## Data Availability

The data used to support the findings of this study are included within the article.

## References

[1] Garimella M. G., Kour S., Piprode V. (2015). Adipose-Derived mesenchymal stem cells prevent systemic bone loss in collagen-induced arthritis. *The Journal of Immunology*.

[2] Wang Y. J., Xu N. L., Zhao S. (2019). miR-410-3p suppresses cytokine release from fibroblast-like synoviocytes by regulating NF-*κ*B signaling in rheumatoid arthritis. *Inflammation*.

[3] Wang Y. J., Jiao T., Fu W. (2019). miR-410-3p regulates proliferation and apoptosis of fibroblast-like synoviocytes by targeting YY1 in rheumatoid arthritis. *Biomedicine & Pharmacotherapy*.

[4] Tu J., Hong W., Zhang P., Wang X., Korner H., Wei W. (2018). Ontology and function of fibroblast-like and macrophage-like synoviocytes: how do they talk to each other and can they Be targeted for rheumatoid arthritis therapy?. *Frontiers in Immunology*.

[5] Kong Q. Z., Guo L. T., Yang J. N. (2016). Anti-inflammatory effects of TRAF-interacting protein in rheumatoid arthritis fibroblast-like synoviocytes. *Mediators of Inflammation*.

[6] Huang W., Shi Y., Han B. (2020). LncRNA GAS5-AS1 inhibits glioma proliferation, migration, and invasion via miR‐106b-5p/TUSC2 axis. *Human Cell*.

[7] Wang N., Feng T., Liu X., Liu Q. (2020). Curcumin inhibits migration and invasion of non-small cell lung cancer cells through up-regulation of miR-206 and suppression of PI3K/AKT/mTOR signaling pathway. *Acta Pharmaceutica*.

[8] Kim J., Abdelmohsen K., Yang X. (2016). LncRNA OIP5-AS1/cyrano sponges RNA-binding protein HuR. *Nucleic Acids Research*.

[9] Zhang J., Zhao T., Tian L., Li Y. (2019). LncRNA OIP5-AS1 promotes the proliferation of hemangioma vascular endothelial cells via regulating miR-195-5p/NOB1 Axis. *Frontiers in Pharmacology*.

[10] Wang L.-W., Li X.-B., Liu Z., Zhao L.-H., Wang Y., Yue L. (2019). Long noncoding RNA OIP5-AS1 promotes proliferation of gastric cancer cells by targeting miR-641. *European Review for Medical and Pharmacological Sciences*.

[11] Song L., Zhou Z., Gan Y. (2019). Long noncoding RNA OIP5‐AS1 causes cisplatin resistance in osteosarcoma through inducing the LPAAT*β*/PI3K/AKT/mTOR signaling pathway by sponging the miR‐340‐5p. *Journal of Cellular Biochemistry*.

[12] Sun W.-Li, Kang T., Wang Y.-Yu (2018). Long Noncoding RNA OIP5-AS1 targets Wnt-7b to affect glioma progression via modulation of miR-410. *Bioence Reports*.

[13] Chen Y., Miao Y., Huang L. (2014). Antioxidant activities of saponins extracted from Radix Trichosanthis: an in vivo and in vitro evaluation. *BMC Complementary and Alternative Medicine*.

[14] Wang Y., Liu Q., Jiang H., Han Y., Li Y. (2016). Metabolomics study in rat urine of adjuvant arthritis using gas chromatography-time-of-flight mass spectrometry. *Chinese Journal of Chromatography*.

[15] Lee E. B., Fleischmann R., Hall S. (2014). Tofacitinib versus methotrexate in rheumatoid arthritis. *New England Journal of Medicine*.

[16] Luo C., Liang J. S., Gong J. (2018). miRNA-31 over-expression improve synovial cells apoptosis induced by RA. *Bratislava Medical Journal*.

[17] Kragstrup T. W., Sohn D. H., Lepus C. M. (2019). Fibroblast-like synovial cell production of extra domain A fibronectin associates with inflammation in osteoarthritis. *BMC Rheumatology*.

[18] Wang W., Guo P., Chen M., Chen D., Cheng Y., He L. (2020). FOXM1/LINC00152 feedback loop regulates proliferation and apoptosis in rheumatoid arthritis fibroblast-like synoviocytes via Wnt/*β*-catenin signaling pathway. *Bioscience Reports*.

[19] Madav Y., Barve K., Prabhakar B. (2020). Current trends in theranostics for rheumatoid arthritis. *European Journal of Pharmaceutical Sciences*.

[20] Demizio D. J., Geraldino-Pardilla L. B. (2020). Autoimmunity and inflammation link to cardiovascular disease risk in rheumatoid arthritis. *Rheumatology and Therapy*.

[21] Zhang X., Wang W., Zhu W. (2019). Mechanisms and functions of long non-coding RNAs at multiple regulatory levels. *International Journal of Molecular Sciences*.

[22] Yang N., Chen J., Zhang H. (2017). LncRNA OIP5-AS1 loss-induced microRNA-410 accumulation regulates cell proliferation and apoptosis by targeting KLF10 via activating PTEN/PI3K/AKT pathway in multiple myeloma. *Cell Death & Disease*.

[23] Zhang Y. F., Yu Y., Song W. Z. (2016). miR-410-3p suppresses breast cancer progression by targeting Snail. *Oncology Reports*.

[24] Li F., Li F., Chen W. (2020). Propofol inhibits cell proliferation, migration, and invasion via mir-410-3p/transforming growth factor-*β* receptor type 2 (TGFBR2) Axis in glioma. *Medical Science Monitor*.

[25] Liu D., Zhang N., Zhang X., Qin M., Dong Y., Jin L. (2016). MiR-410 down-regulates the expression of interleukin-10 by targeting STAT3 in the pathogenesis of systemic lupus erythematosus. *Cellular Physiology and Biochemistry*.

[26] Huang T., Zhou Y., Wang J., Cao Y., Hang D. H. (2019). MiR-26b regulates cartilage differentiation of bone marrow mesenchymal stem cells in rats through the Wnt/*β*-catenin signaling pathway. *European Review for Medical and Pharmacological Sciences*.

[27] Maeda K., Takahashi N., Kobayashi Y. (2013). Roles of Wnt signals in bone resorption during physiological and pathological states. *Journal of Molecular Medicine*.

[28] Xiao C. Y., Pan Y. F., Guo X. H., Wu Y. Q., Gu J. R., Cai D. Z. (2010). Expression of *β*-catenin in rheumatoid arthritis fibroblast-like synoviocytes. *Scandinavian Journal of Rheumatology*.

[29] Yoshioka R., Kita Y., Nagahira A. (2015). Quantitative analysis of cadherin-11 and -catenin signalling during proliferation of rheumatoid arthritis-derived synovial fibroblast cells. *Journal of Pharmacy and Pharmacology*.

[30] Zhang Z., Liu M., Hu Q. (2019). FGFBP1, a downstream target of the FBW7/c-Myc axis, promotes cell proliferation and migration in pancreatic cancer. *American Journal of Cancer Research*.

[31] Faccidomo S., Holstein S. E., Santanam T. S. (2020). Pharmacological inhibition of glycogen synthase kinase 3 increases operant alcohol self-administration in a manner associated with altered pGSK-3*β*, protein interacting with C kinase and GluA2 protein expression in the reward pathway of male C57BL/6J mice. *Behavioural Pharmacology*.

[32] Reabroi S., Saeeng R., Boonmuen N. (2018). The anti-cancer activity of an andrographolide analogue functions through a GSK-3*β*-independent Wnt/*β*-catenin signaling pathway in colorectal cancer cells. *Scientific Reports*.

[33] Miao C. G., Huang C., Huang Y. (2013). MeCP2 modulates the canonical Wnt pathway activation by targeting SFRP4 in rheumatoid arthritis fibroblast-like synoviocytes in rats. *Cellular Signalling*.

